# Critical role of bevacizumab scheduling in combination with pre-surgical chemo-radiotherapy in MRI-defined high-risk locally advanced rectal cancer: results of the branch trial

**DOI:** 10.18632/oncotarget.4724

**Published:** 2015-07-30

**Authors:** Antonio Avallone, Biagio Pecori, Franco Bianco, Luigi Aloj, Fabiana Tatangelo, Carmela Romano, Vincenza Granata, Pietro Marone, Alessandra Leone, Gerardo Botti, Antonella Petrillo, Corradina Caracò, Vincenzo R. Iaffaioli, Paolo Muto, Giovanni Romano, Pasquale Comella, Alfredo Budillon, Paolo Delrio

**Affiliations:** ^1^ Gastrointestinal Medical Oncology Unit, Istituto Nazionale per lo Studio e la Cura dei Tumori “Fondazione Giovanni Pascale” – IRCCS, 80131, Napoli, Italy; ^2^ Radiotherapy Unit, Istituto Nazionale per lo Studio e la Cura dei Tumori “Fondazione Giovanni Pascale” – IRCCS, 80131, Napoli, Italy; ^3^ Gastrointestinal Surgery, Istituto Nazionale per lo Studio e la Cura dei Tumori “Fondazione Giovanni Pascale” – IRCCS, 80131, Napoli, Italy; ^4^ Nuclear Medicine Unit, Istituto Nazionale per lo Studio e la Cura dei Tumori “Fondazione Giovanni Pascale” – IRCCS, 80131, Napoli, Italy; ^5^ Pathology Unit, Istituto Nazionale per lo Studio e la Cura dei Tumori “Fondazione Giovanni Pascale” – IRCCS, 80131, Napoli, Italy; ^6^ Radiology Unit, Istituto Nazionale per lo Studio e la Cura dei Tumori “Fondazione Giovanni Pascale” – IRCCS, 80131, Napoli, Italy; ^7^ Endoscopy Unit, Istituto Nazionale per lo Studio e la Cura dei Tumori “Fondazione Giovanni Pascale” – IRCCS, 80131, Napoli, Italy; ^8^ Experimental Pharmacology Unit, Istituto Nazionale per lo Studio e la Cura dei Tumori “Fondazione Giovanni Pascale” – IRCCS, 80131, Napoli, Italy; ^9^ Colorectal Surgery Unit, Istituto Nazionale per lo Studio e la Cura dei Tumori “Fondazione Giovanni Pascale” – IRCCS, 80131, Napoli, Italy

**Keywords:** Clinical Section, locally advanced rectal cancer, bevacizumab, preoperative chemo-radiotherapy, adjuvant chemotherapy, vessel normalization

## Abstract

**Background:**

We have previously shown that an intensified preoperative regimen including oxaliplatin plus raltitrexed and 5-fluorouracil/folinic acid (OXATOM/FUFA) during preoperative pelvic radiotherapy produced promising results in locally advanced rectal cancer (LARC). Preclinical evidence suggests that the scheduling of bevacizumab may be crucial to optimize its combination with chemo-radiotherapy.

**Patients and methods:**

This non-randomized, non-comparative, phase II study was conducted in MRI-defined high-risk LARC. Patients received three biweekly cycles of OXATOM/FUFA during RT. Bevacizumab was given 2 weeks before the start of chemo-radiotherapy, and on the same day of chemotherapy for 3 cycles (concomitant-schedule A) or 4 days prior to the first and second cycle of chemotherapy (sequential-schedule B). Primary end point was pathological complete tumor regression (TRG1) rate.

**Results:**

The accrual for the concomitant-schedule was early terminated because the number of TRG1 (2 out of 16 patients) was statistically inconsistent with the hypothesis of activity (30%) to be tested. Conversely, the endpoint was reached with the sequential-schedule and the final TRG1 rate among 46 enrolled patients was 50% (95% CI 35%–65%). Neutropenia was the most common grade ≥3 toxicity with both schedules, but it was less pronounced with the sequential than concomitant-schedule (30% vs. 44%). Postoperative complications occurred in 8/15 (53%) and 13/46 (28%) patients in schedule A and B, respectively. At 5 year follow-up the probability of PFS and OS was 80% (95%CI, 66%–89%) and 85% (95%CI, 69%–93%), respectively, for the sequential-schedule.

**Conclusions:**

These results highlights the relevance of bevacizumab scheduling to optimize its combination with preoperative chemo-radiotherapy in the management of LARC.

## INTRODUCTION

The evolution of preoperative multimodality treatment has improved the local control of locally advanced rectal cancer (LARC). Distant metastases, however, remain a clinical challenge and more effective systemic approaches are needed [1]. LARC comprises a heterogeneous group of tumors in which outcomes vary significantly depending on prognostic factors, namely T and N stage, involvement of the circumferential resection margin (CRM) and low-lying location [2]. The high-resolution magnetic resonance imaging (MRI) allows the preoperative identification of high-risk features, enabling patient selection for risk-adapted treatment [2]. In such high-risk LARC patients we have recently reported promising results from a phase-2 trial based on an original intensified preoperative chemotherapy (CT) regimen including oxaliplatin plus raltitrexed and 5-fluorouracil modulated by folinic acid (OXATOM/FUFA) at full systemic doses, during preoperative pelvic radiotherapy (RT). High rates of complete (40%) and near-complete (25%) pathologic tumor regression were observed, with a 5-year freedom from recurrence of 80% and a 5-year overall survival (OS) of 87% [3]. On the basis of preclinical and clinical data the integration of bevacizumab into fluoropyrimidines-based chemo-radiotherapy (CRT) has been extensively investigated in phase II studies [4, 5]. However, results overall have been modest and concerns regarding increased surgical morbidity have been raised [6]. Several evidences suggest that the chemo-sensitizing activity exerted by anti-VEGF agents depends on the so-called “vessel to normalization, in which a reduction of tumor vessel abnormalities results in more efficient delivery of drugs and oxygen to cancer cells [7]. Although genetic studies have shown prolonged maintenance of vascular normalization [8], in preclinical models pharmacological VEGF inhibitors induced a transient vessel normalization, occurring a few days after anti-VEGF administration [9, 10]. Therefore, rescheduling of bevacizumab relative to CT and RT could be of critical importance to optimize the efficacy of the combination treatment. On these basis we conducted a phase-2 study in MRI-defined high-risk LARC patients, in order to evaluate the addition of bevacizumab to OXATOM/FUFA during preoperative RT.

A concomitant schedule, in which bevacizumab was administered concurrentlyy to CT, was initially evaluated. Thereafter, a sequential schedule, in which bevacizumab was given 4 days before CT, was also evaluated in order to explore the clinical relevance of “vessel normalization”.

The primary endpoint of the study was the rate of TRG1. However, considering the controversial prognostic value of short term pathological end points and the paucity of data on long-term outcomes of LARC patients, we also report on these findings with a prolonged follow-up.

## RESULTS

### Patient characteristics and treatment

Between December 2006 and July 2011 16 patients were enrolled in schedule A (concomitant) and 46 in schedule B (sequential). Demographic and baseline disease characteristics by treatment schedule are shown in Table 1. Most patients had more than one high-risk factor. Among patients with radial margin ≤5 mm from the mesorectal fascia (MRF), evaluated by MRI and considered to have potentially positive CRM, 4/13 (31%) in schedule A and 29/35 (83%) in schedule B showed tumor location within 2 mm of the MRF. All T3N0-1 tumors had a potentially positive CRM and/or extramural extension ≥5 mm.

**Table 1:** Baseline patient and tumor characteristics

Characteristics	Schedule A *n* = 16 (% of total)	Schedule B *n* = 46 (% of total)
Gender		
Male/Female	9 (56)/7 (44)	28 (61)/18 (39)
Median age (range)	55 (48–69)	61 (43–74)
ECOG Performance status		
0	8 (50)	22 (48)
1/2	7 (44)/ 1 (6)	13 (50)/1 (2)
Clinical staging		
T3N0	3 (19)	1 (2)
T3N1/T4N0	7 (44)/1 (6)	14 (30)/1 (2)
T3N2/T4N1-2	2 (12)/2 (12)	22 (48)/1 (2)
T3N0M1/T3N1M1	–	1 (2)/2 (4)
T3N2M1/T4N2M1	1 (6)/0	3 (7)/1 (2)
Distance from the anal verge		
≤5 cm (low-lying tumor)	9 (56)	25 (54)
>5 cm (mid/upper tumor)	7 (44)	21 (46)
Distance of Mesorectal Fascia (MRF)		
≤5 mm	13 (81)	35 (76)
>5 mm	2 (12)	8 (18)
Not evaluated*	1 (6)	3 (7)
Baseline CEA serum level		
≤5 UI/L	10 (62)	28(61)
>5 UI/L	6 (37)	18 (39)

* MRI not performed, metal prosthesis.

All patients in schedule A and all but 1 patient in schedule B received the planned dose of RT. The radiation treatment was completed in a median of 39 (range, 35–45) and 36 (range, 33–49) days in schedule A and B, respectively. One patient in each group did not receive the third cycle of chemotherapy due to severe and persistent neutropenia and diarrhea. Overall, the median relative dose intensities of cytotoxic drugs were: 87% and 97% for oxaliplatin, 87% and 97% for raltitrexed, 83% and 92% for 5-FU, 94% and 100% for bevacizumab, in schedule A and B, respectively. The only patient with metastatic disease enrolled in schedule A and 4 out of 7 metastatic patients in schedule B received two additional cycles of chemotherapy after the end of RT. One patient in schedule A with a complete clinical response refused surgery. The median interval between the end of RT and TME was 9 weeks for both schedule A (range, 7–14) and B (range, 7–15).

### Outcome

Surgical outcome and pathological tumor responses are summarized in Table 2. Notably, the incomplete resection in schedule B pertained only to metastatic disease. There were no instances of disease progression during treatment.

**Table 2:** Surgical outcomes and pathological tumor response

Parameters	Schedule A^°^(*n* = 16) *n* (%; 95% CI)	Schedule B (*n* = 46) *n* (%; 95% CI)
Surgery type		
Anterior resection	13 (81;57–93)	41 (89;77–95)
Abdominoperineal resection	2 (12;3–36)	5 (11;5–23)
Sphincter preservation in patients with tumor ≤5 cm from anal verge	5/9 (56;27–81)	20/25 (80;61–91)
Resection Status		
Complete resection (R0)	13 (81;57–93)	43 (93;82–98)
Microscopic residual disease (R1)	1* (6;1–28)	1** (2;0–11)
Macroscopic residual disease (R2)	1* (6;1–28)	2** (4;1–14)
ypTypN status		
ypT0ypN0 (pCR)	2 (12;3–36)	19 (41;28–56)
ypT0-2ypN0	8 (50;28–72)	30 (65;51–77)
ypN1-2	5 (31;14–56)	14 (30;19–45)
Mandard tumor regression grade		
TRG1	2 (12;3–36)	23 (50;36–64)
TRG2	8 (50;28–72)	15 (33;21–47)
TRG3	3 (19;7–43)	7 (15;7–28)
TRG4	2 (12;3–36)	1 (2;0–11)
TRG5	0	0

° one patient refused surgery;

* R1 or R2 resection on primary tumor;

** R1 or R2 resection on metastatic disease

According to the preplanned first-stage analysis, the accrual for schedule A was terminated since only 2 TRG1 responses were identified. Conversely, in schedule B, 8/15 patients showed a TRG1 in the first stage and 23/46 (50%; 95% CI, 35%–65%) in the second stage. Importantly, TRG1 status was achieved independently of the pre-treatment clinical stage (Table 3). In addition, 15 patients (33%; 95% CI, 21%–47%) obtained a near complete tumor regression with schedule B (TRG2, Table 2). Pathological tumor downstaging occurred in 39/46 (85%) patients (Table 3).

**Table 3:** Comparison of baseline staging with pathological findings in schedule B

	Tumor Regression Grade (TRG)			
	Schedule B *n* = 46			
	TRG1	TRG2	TRG3	TRG4
Baseline Staging	*n* = 23	*n* = 15	*n* = 7	*n* = 1
cT3N0 *n* = 1		1(ypT2N0)		
cT3N1 *n* = 14	6(ypT0N0); 1(ypT0N1b)	2(ypT2N0) 1(ypT2N1a)	1(ypT2N0);1(ypT3N0) 1(ypT2N1a)	1(ypT3N2b)
T4N0 *n* = 1	1(ypT0N0)			
cT3N2 *n* = 22	7(ypT0N0); 1(ypT0N1b)	3(ypT1N0);3(ypT2N0) 1(ypT2N1a);2(ypT2N1b) 1(ypT2N2a)1(ypT3N2a)	1(ypT1N0);1(ypT3N0) 1(ypT3N1a)	
cT4N1-2 *n* = 1	1(ypT0N0)			
cT3N0M1 *n* = 1	1(ypT0N0cM0)*			
cT3N1M1 *n* = 2	1(ypT0N0M1) 1(ypT0N1aM1)			
cT3N2M1 *n* = 3	1(ypT0N0M0) 1(ypT0N2bM0)		1(ypT2N2aM1)	
cT4N2M1 *n* = 1	1(ypT0N0M1)			

cTNM = clinical staging; ypTNM = pathological staging after chemoradiotherapy treatment

* clinical complete response of the lung metastases

A median of 32 (range, 19–62) and 27 (range, 12–108) lymph nodes per patient were harvested in schedule A and B, respectively. Pathological nodal downstaging occurred in 32/43 (74%) patients (Table 3). In schedule B, 4 patients with TRG1 (2 with baseline metastatic disease) revealed pathological nodal involvement. Overall pCR rate was 41% and 30/46 (65%) patients had a ypT0–2N0 status (Table 2).

As provided by the selective adjuvant policy adopted in the study, 5 patients in schedule A and 13/16 patients in schedule B (3 patients with baseline metastatic disease and R0/R1 resection refused further treatment), received post-operative FOLFOX4 after a median of 7 weeks (schedule A, range: 7–9; schedule B, range: 4–15) from surgery. In schedule A, with a median follow-up at the time of analysis of 91 (range, 84–96) months, 2 patients had distant recurrence, 3 patients had both local and distant recurrence and 5 patients died.

**Figure 1:**
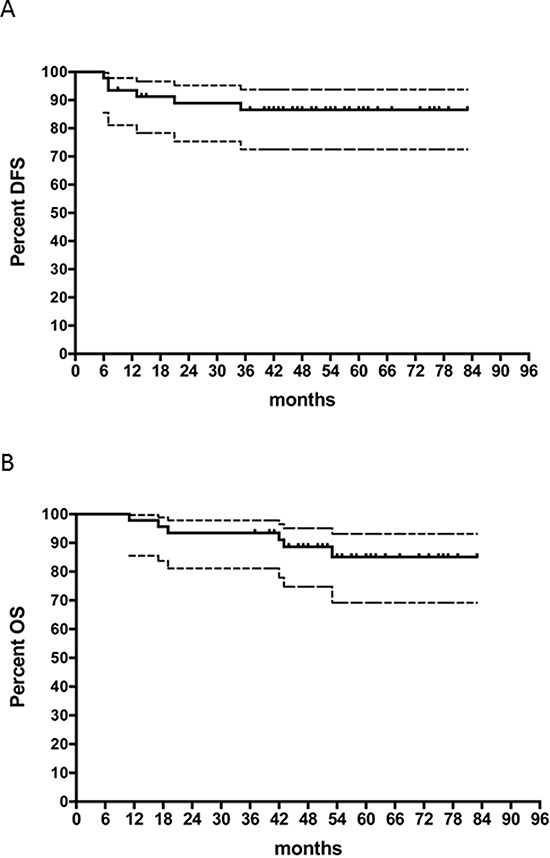
Kaplan-Meier survival curves Dashed curves represent 95% CIs. **A.** Progression-free survival for the schedule B. **B.** Overall survival for the schedule B.

In schedule B after a median follow up of 54 (range, 41–83) months, 37/46 (80%) patients continued to be disease-free. One patient (ypN+) had a local recurrence, 3 patients (including 1 with early interruption of CRT and 1 ypN+) had both local and distant recurrences and 2 patients (both ypN+) had distant recurrence. Of note, 4 patients with baseline metastatic disease (3 achieving a TRG1 response) were still disease-free. Six patients died of rectal cancer specific causes. At 5-years follow-up the probability of Progression-free survival (PFS) and OS was 80% (95%CI, 66%-89%) and, 85% (95%CI, 69%-93%), respectively, for schedule B (Figure 1).

### Safety

Treatment induced side effects are summarized in Table 4. Toxicity profiles were similar between the two schedules and neutropenia was the most common grade ≥3 adverse event, but it was short lasting and easily managed. Notably, severe neutropenia was less pronounced in schedule B (30% vs. 44%). Furthermore, 6/14 (43%) episodes of severe neutropenia in schedule B, as opposed to 1/7 (14%) in schedule A, occurred only after the end of CRT, thus not causing treatment delays or dose modifications. No cardiac or thromboembolic events, hemorrhages or perforations were observed.

**Table 4:** Treatment Related Toxicity

	Schedule A (*n* = 16)						Schedule B (*n* = 46)					
Toxicity	Grade 1–2		Grade 3–4		Total		Grade 1–2		Grade 3–4		Total	
	No.	%	No.	%	No.	%	No.	%	No.	%	No.	%
Neutropenia	7	44	7	44	14	87	9	20	14	30	23	50
Thrombocytopenia	1	6	0		1	6	1	2	1	2	2	4
Anemia	0		1	6	1	6	6	13	1	2	7	15
Nausea/vomiting	7	44	0		7	44	22	48	0		22	48
Abdominal pain	3	19	0		3	19	4	9	0		4	9
Diarrhea	6	37	1	6	7	43	16	35	3	6	19	41
Stomatitis	1	6	0		1	6	2	4	0		2	4
Proctitis	4	25	0		4	25	14	30	0		14	30
Proteinuria	3	19	0		3	19	4	9	0		4	9
Liver enzymes	2	12	0		2	12	9	20	1	2	10	22
Skin reactions	3	19	0		3	19	5	11	0		5	11
Asthenia	4	25	0		4	25	10	22	2	4	12	26
Neuropathy	2	12	0		2	12	6	13	0		6	13
Hypertension	4	25	1	6	5	31	11	24	0		11	24
Anorexia	2	12	0		2	12	3	6	2	4	5	10
Rectal tenesmus	2	12	0		2	12	6	13	0		6	13
Cystitis	2	12	0		2	12	4	9	0		4	9

Postoperative complications occurred in 8/15 (53%) and 13/46 (28%) patients in schedule A and B, respectively (Table 5). Re-operation was necessary in 3 patients (19%) in schedule A and in 4 patients (9%) in schedule B. One patient per schedule required a permanent colostomy. There were no deaths correlated with CRT or surgery in either treatment schedules.

**Table 5:** Postoperative Complications and late toxicities

	Schedule A (*n* = 16)	Schedule B (*n* = 46)
**Patients with at least one complication, *n* (%)**	8 (53%)	13 (28%)
Anastomotic fistula	3	3
Rectovaginal fistula	1	−
Pelvic infection	2	5
Anastomotic leak	1	2
Intestinal ischemia	1	–
Wound healing complication	–	1
Urinary retention	–	3
Rectal bleeding	–	1
Stoma complication	–	1
Anastomotic stenosis	–	2
		
**Patients with at least one late toxicity, *n* (%)**	6 (40%)	19 (41%)
Sexual dysfunction		
Erectile dysfunction	2	4
Retrograde ejaculation	–	1
Dyspareunia	1	1
Anorectal dysfunction		–
Fecal and gas incontinence	2	7
Higher stool frequency	2	5
Constipation	–	1
Urinary dysfunction		1
Neurogenic bladder	–	1

Moderate or severe late toxicities were reported in 6/15 (40%) and 19/46 (41%) patients in schedule A and B, respectively (Table 5).

## DISCUSSION

The present results support the hypothesis that rescheduling of anti-VEGF treatment relative to CT and RT could be of critical importance to improve the efficacy of combination treatment. The concomitant delivery of bevacizumab with the OXATOM/FUFA regimen during RT did not meet the primary endpoint.

With the sequential schedule, on the other hand, the endpoint was reached in both stages of Simon's statistical design and the final TRG1 rate was 50% (95% CI, 35%–65%), slightly better compared to our previous experience with the same regimen without bevacizumab (40%) [3]. In addition, a near complete tumor regression was observed in 15 further patients, with an overall rate of TRG1/TRG2 responses of 83% (95% CI, 69%–91%), which was higher compared to our previous experience without bevacizumab (65%) [3]. Moreover, we should also point out that most patients enrolled in the current study had more than one high-risk factor, including 15% of patients with metastatic disease, who were not included in our previous experience.

Although four patients with TRG1 had pathological nodal involvement, the 41% pCR rate is still impressive. Notably, these results were observed regardless of pretreatment clinical stage and were reported through a robust pathological analysis, as proven by the elevated median number of lymph nodes retrieved. Additional remarkable findings were the 93% R0 resection rate, the 65% ypT0-2N0 rate, the 80% sphincter-sparing surgery rate and the clinical or pathological complete response of metastatic sites in 3/7 patients. All this data endorses the efficacy of the sequential bevacizumab schedule with OXATOM/FUFA during RT. The consistency of these findings was corroborated by the long-term outcome of patients.

Indeed, the 80% 5-year PFS and 85% 5-year OS indicates a good and durable distant control. These results are particularly relevant considering the poor prognostic features of the enrolled patients.

The selective adjuvant policy adopted in our trial strengthens the value of these findings, minimizing the influence of the adjuvant therapy on long-term outcome. Indeed, postoperative FOLFOX4 was delivered only in a small subgroup of patients. Although there is no general agreement on the benefit of adjuvant chemotherapy after preoperative CRT [11], there is recent compelling evidence supporting the use of a risk-adapted strategy based on pathological findings to select candidates for CT after surgery [12, 13]. Moreover, recent results of the randomized phase-2 ADORE study in LARC patients suggest that those less responsive to fluoropyrimidine-based CRT may benefit from four months of adjuvant FOLFOX chemotherapy [14].

Although comparisons across studies should be made with caution, our findings are encouraging when compared to results of similar studies employing preoperative treatment on MRI-defined high-risk LARC patients [15]. Only two studies investigating the integration of bevacizumab into preoperative treatment reported a pCR rate >30% (Table 6 [16–28]).

**Table 6:** Main published phase II studies using bevacizumab in combination with preoperative radiochemotherapy

Author	Reference	Treatment schedule	Eligible patients	Main Toxicity G3/G4 (%)	Postoperative complications* (%)	Complete tumor regression** (%)	Survival
Willett et al.	[14, 15]	RT 50.4 Gy; BEV days−14, 1, 15, 29; 5FU 225 mg/m^2^/d days 1–38	T3–T4 (*n* = 32)	Diarrhea 22	Minor 28 Major 6	5/32 (16)	5-years DFS 77% 5-years OS 100%
Crane et al.	[16]	RT 50.4 Gy; BEV days 1, 15, 29; Cap 900 mg/m^2^ b.i.d. days 1–38	T3–T4 and/or N+ (*n* = 25)	0	Minor 20 Major 12	8/25 (32)	2-years DFS 69% 2-years OS 95%
Noguè et al.	[17]	Induction: BEV+XELOX × 4; RT 50.4 Gy; BEV days 1, 15, 29; Cap 825 mg/m^2^ b.i.d. days 1–38	MRI-defined high-risk T3–T4 and/or N+ (*n* = 47)	Diarrhea 11 Neutropenia 6	Minor 34 Major 24	16/47 (34)	NS
Velenik et al.	[18]	RT 50.4 Gy; BEV days−14, 1, 15, 29; Cap 825 mg/m^2^ b.i.d. days 1–38	T3–T4 and/or N+ (*n* = 61)	Dermatitis 10	Minor 52 Major 10	8/61 (13)	NS
Dipetrillo et al.	[19]	Induction: BEV+FOLFOX × 2; RT 50.4 Gy; BEV days 1, 15, 29; 5FU200 mg/m^2^/d days 1–38; OX 40 mg/m^2^ weekly × 6 weeks	T3–T4 and/or N+ (*n* = 26)	Diarrhea 44 Neutropenia 20	Minor 36	5/26 (19)	3-years DFS 65% 3-years OS 95%
Gasparini et al.	[20]	RT 50.4 Gy; BEV days−14, 1, 15, 29; Cap 825 mg/m^2^ b.i.d. days 1–38	T3–T4 and/or N+ (*n* = 43)	Diarrhea 7	Minor 7 Major 2°	6/43 (14)	3-years DFS 75%
Kennecke et al.	[21]	RT 50.4 Gy; BEV days−14, 1, 15, 29; Cap 825 mg/m^2^ b.i.d. days 1–14 and 22–35; OX 50 mg/m^2^ days 1, 8, 22, and 29	High-risk T3–T4 and/or N+ or anyTNM1 (*n* = 42)	Diarrhea 24 Rectal pain 10	Minor 53 Major 10	9/42 (21)	NS
Spigel et al.	[22]	RT 50.4 Gy; BEV days 1, 15; 5FU 225 mg/m^2^/d days 1–42	T3–T4 and/or N+ (*n* = 35)	Thrombocytopenia 9 Diarrhea 6	Minor 3 Major 3	10/35 (29)	1-years DFS 85%
Landry et al.	[23]	RT 50.4 Gy; BEV days 1, 15, 29; Cap 825 mg/m^2^ b.i.d. days 1–38; OX 50 mg/m^2^ weekly × 5 weeks	T3–T4 (*n* = 54)	Rectal pain 17 Diarrhea 13^§§^	Minor 49 Major 6	9/54 (17)	NS
Dellas et al.	[24]	RT 50.4 Gy; BEV days 1, 15, 29; Cap 825 mg/m^2^ b.i.d. days 1–14 and 22–35; OX 50 mg/m^2^ days 1, 8, 22, and 29	T3–T4 and/or N+ or any TNM 1livrs (*n* = 69)	Diarrhea 4	Minor 55	12/69 (17)	NS
Blaszkowsky et al.	[25]	RT 50.4 Gy; BEV days −14, 1, 15, 29; 5FU 225 mg/m^2^/d days 1–38; erlo 100 mg/m^2^/d days 1–38	T3–T4 (*n* = 26)	Lymphopenia 46 Diarrhea 19	Minor 65	7/26 (27)	3-years DFS 75%
Borg et al.	[26]	Induction: BEV+FOLFOX × 6; RT 45 Gy; BEV days−14, 1, 15, 29; 5FU 225 mg/m^2^/d × 5 days/week	MRI-defined locally advanced T3 (*n* = 46)	Neutropenia 20 Diarrhea 6 Gastrointestinal perforation 4	Minor 22	10/46 (22)	NS
Borg et al.	[26]	RT 45 Gy; BEV days−14, 1, 15, 29; 5FU 225 mg/m^2^/d × 5 days/week	MRI-defined locally advanced T3 (*n* = 45)	Diarrhea 6 Proctitis 4	Minor 22	5/45 (11)	NS
Present study Schedule B		RT 45 Gy; BEV days−4, 11; OX 100 mg/m^2^ + Tom 2.5 mg/m^2^ days 1, 15 and 29; 5FU 800 mg/m^2^ + LFA 250 mg/m^2^ days 2, 16 and 30	MRI-defined high-risk T3–T4 and/or N+ or any TNM1rs (*n* = 46)	Neutropenia 30 Diarrea 6	Minor 19 Major 9	23/46 (50)	5-years PFS 80% 5-years OS 85%

RT = radiotherapy; BEV = bevacizumab; 5-FU = 5-fluorouracil; Cap = capecitabine; OX = oxaliplatin; LFA = folinic acid; erlo = erlotinib; d = day; .livrs = single resectable liver metastasis; rs = resectable; MRI = magnetic resonance imaging; NS = not specified. DFS = disease free survival; PFS = progression free survival; OS = overall survival

* Postoperative surgery-related complications Major/minor requiring/not requiring surgical reintervention or drainage

** Various tumor regression grading systems used

° One postoperative death

§ Two (4%) toxicity-related deaths (one sudden death and one ketoacidosis)

§§ One toxicity-related death (aspiration)

Crane and colleagues [18] reported a pCR rate of 32% and an additional microscopic residual disease rate of 24% with the administration of capecitabine and bevacizumab concomitant to preoperative RT. However, patient selection in this study, as with most studies assessing bevacizumab, was not based on MRI criteria (Table 6 [16–28]). Furthermore, PFS (with a shorter follow-up) was markedly lower than in our series [18]. A slightly higher pCR rate of 34% and an additional near-ypCR rate of 36% were observed in a phase-2 study, in which patients received induction chemotherapy with 4 cycles of bevacizumab plus XELOX, followed by CRT with concurrent capecitabine and bevacizumab. In this study, MRI selection criteria were used but, unlike in our study, patients with metastatic disease were not included, dose of RT was higher and long-term outcome was not reported [19].

With respect to safety, the combination of bevacizumab with RT and OXATOM/FUFA was well tolerated. Toxicities were manageable and similar to those previously reported with the same CRT regimen without bevacizumab [3]. Neutropenia was the most common toxicity but did not decrease the treatment compliance. Interestingly the incidence of grade ≥3 neutropenia reported for schedule B was lower compared to the same CRT regimen without or with bevacizumab in the concomitant schedule. In line with these results, recent preclinical data shows that the sequential delivery of antiangiogenic drugs followed by chemotherapy yields a lower bone marrow toxicity compared to the concomitant administration [29].

In agreement with some trials evaluating the integration of bevacizumab in preoperative treatment (Table 5), we observed high a rate of surgical complications and re-operation under schedule A, while postoperative complications in schedule B were relatively low (Table 5). We hypothesize that the administration of only two cycles of bevacizumab, and the resulting longer interval between its last administration and surgery, may have helped to reduce the occurrence of surgical complications. However, compared to the 2% rate of re-operation reported in larger phase III trial in patients treated with preoperative fluoropyrimidine alone during RT [30], the 9% rate observed with the sequential schedule is higher. Nevertheless, this rate is similar to that reported with other intensified treatment approaches, including our previous experience without bevacizumab [3, 31]. Notably when an intensified approach was used in combination with bevacizumab as preoperative treatment, an even higher re-operation rate was reported [19].

Our study has some potential limitations that deserve special consideration. Its major weakness is that it was a single center, non-randomized phase-2 trial, with a relatively small sample size. Furthermore, evidence is emerging that a margin of ≤1 mm from the MRF by MRI may be adequate to identify patients at risk of CRM involvement [32], as opposed to the 5 mm threshold previously accepted [33]. A recent multicenter study showed that a cut-off of 2 mm from the MRF may help select patients in whom an intensive preoperative treatment is needed [34]. In our study, the tumor was located within 2 mm of MRF in 83% of patients and most patients had additional high-risk factors. Moreover, 93% of patients showed clinical lymph node involvement and 15% had metastatic disease. Therefore, it seems unlikely that our results were influenced by favorable patient selection.

In conclusion, taking into account the few doses of bevacizumab associated with preoperative CRT, its scheduling seems to be crucial to potentiate the combination. We may hypothesize that, in this setting, bevacizumab might improve the delivery of cytotoxic drugs and oxygen to cancer cells through a reduction of the interstitial fluid pressure (“vessel normalization effect”), that has been proven to occur few days after a single drug administration [5], rather than through the reduction of vessel density (“tumor-starving effect”). If so, the sequential administration of bevacizumab and CRT, as opposed to concurrent, exploits this synergism at best. Of course, further studies are needed to validate this approach and better understand its underlying molecular mechanisms. Therefore, we are currently conducting a phase-3 randomized study comparing the two bevacizumab schedules in combination with FOLFOX/OXXEL regimens in metastatic colorectal cancer patients (OBELICS study, NCT01718873).

## MATERIALS AND METHODS

The BRANCH (Bevacizumab, RAdiotherapy aNd CHemotherapy) trial was an open label, non-randomized, single center, phase-2 clinical study (Eudract number: 2008-003989-26; Clinicaltrials.gov number: NCT01481545), approved by the local ethical committee and conducted in accordance to the Declaration of Helsinki. Signed written informed consent was obtained from each patient before accrual.

### Patient selection

The study enrolled patients with pathologically confirmed untreated high-risk rectal adenocarcinoma within 15 cm from the anal margin. High-risk was defined a tumor with concomitant resectable metastases and/or any of the following MRI features: T4; anyTN1-2; T3N0 tumors located in the lower third of the rectum and/or whose radial margin was ≤5 mm from MRF. Tumors with radial margin ≤5 mm from MRF, defined by MRI, were considered to have potentially positive CRM. Other main eligibility criteria were: age ≥18 years; ECOG performance status ≤2; adequate bone marrow, renal, and liver function; absence of concurrent uncontrolled medical condition; no previous malignant disease.

Baseline work-up was completed within 6 weeks before the registration and included: clinical examination, laboratory tests, recto-colonoscopy and endorectal ultrasound; carcinoembryonic antigen (CEA) serum level; whole body contrast enhanced computed tomography (CT); MRI of the pelvis. Imaging studies were blindly reviewed by two radiologists (AP and VG) and in case of discrepancy the worse TNM was assigned.

Biomarker studies on tumor and blood samples and [^18^F]-2-fluoro-2-deoxy-D-glucose positron emission tomography (^18^FDG-PET) evaluation were also performed at baseline, during treatment and before surgery. Results of these studies will be described in separate reports.

### Study Procedures

#### Treatment plan

The total dose of RT was 45 Gy delivered in 25 fractions of 1.8 Gy, 5 days/week. The clinical target volume (CTV) included the tumor, with margins of 2–3 cm depending on tumor direction, mesorectum and regional lymph nodes. The planning target volume was defined as CTV + 1 cm margin.

Chemotherapy given during RT consisted of three biweekly cycles of infusional oxaliplatin, 100 mg/m^2^ followed by raltitrexed, 2.5 mg/m^2^ on day 1, and levo-folinic acid, 250 mg/m^2^ followed by bolus of 5-fluorouracil, 800 mg/m^2^ on day 2.

**Figure 2:**
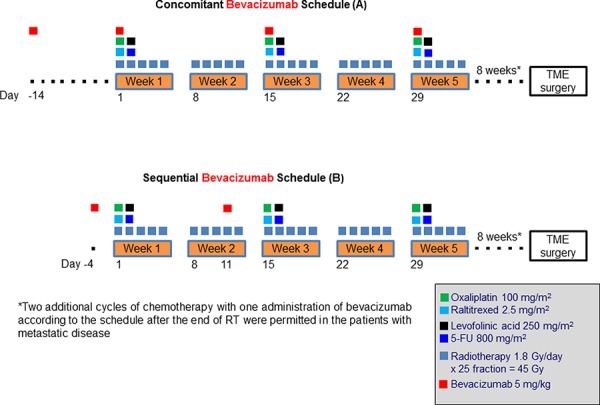
Treatment schedules

Bevacizumab 5 mg/kg was administered 2 weeks before the start of CRT, and on the same day of oxaliplatin and raltitrexed for 3 cycles (treatment days: -14, 1, 15 and 29; concomitant schedule A) or 4 days prior to the first and second cycle of chemotherapy (treatment days: -4, and 11; sequential schedule B) (Figure 2). Two additional cycles of chemotherapy with one bevacizumab infusion, using the previously employed timing schedule, were allowed after the end of CRT in patients with metastatic disease.

CRT had to be temporarily stopped in case of grade ≥3 toxicity. Dose adjustments for adverse events were reported previously [35]. Total mesorectal excision (TME) was planned 8 weeks (±1) after the last day of RT. The choice of surgical procedure for primary tumor, anterior or abdominal–peritoneal resection, and metastases was based on restaging. Fecal diversion to protect the anastomosis was performed by means of a loop ileostomy, which was later reversed after endoscopic assessment of anastomotic integrity. All patients underwent clinical tumor response assessment before surgery with the same imaging modalities that were used for the inclusion into the study.

#### Pathology

Tumor regression grade (TRG) was blindly measured according to the Mandard scale [36] by two pathologists (GB and FT). In case of discrepancy between the two the worse TRG score was assigned.

A complete pathologic response (ypCR,) was defined as the absence of viable tumor cells in the primary tumor and lymph nodes (ypT0N0). Radical resection (R0) was defined as macroscopic and microscopic tumor-free resection for both primary tumor (CRM > 1 mm) and metastatic disease (margins > 1 mm from tumor).

#### Adjuvant treatment and follow up

Four months of post-operative FOLFOX4 regimen, no earlier than 4 weeks after surgery, was planned only in patients with ypN+ or CRM ≤ 1 mm at pathology examination, or for those with baseline metastatic disease eventually resected with an R0/R1 status.

Clinical examination, CEA serum level, whole body CT and pelvic MRI, were performed every 4 months for the first 2 years of follow up, every 6 months for the next 3 years, and annually thereafter.

### Statistical design and analysis

The primary end point of this study was the rate of TRG1. To establish the sample size, the Simon's two-stage design was applied [37]. Setting α and β errors at 0.05 and 0.20, respectively, and defining as the minimum activity of interest (p0) a TRG1 rate of 30%, in order to accept the alternative hypothesis (p1) of a TRG1 rate ≥ 50%, at least 6 TRG1 in the first 15 patients and at least 19 TRG1 among a total of 46 patients would need to be reported in the first and second stage, respectively.

The study protocol provided that the sequential schedule of bevacizumab would be evaluated only if the required number of TRG1 was not reached with the concomitant schedule. Other endpoints included toxicity, surgical morbidity, and long-term outcome.

Toxicities, were graded according to the National Cancer Institute common toxicity criteria (NCI CTC-Version 3). Postoperative complications were included in this report when occurring within 90 days from surgery. Late toxicity was assessed using the SOMA-LENT scale [38]. Proportions were calculated with their exact 95% confidence interval (CI).

PFS was calculated from the date of initial treatment until tumor progression or relapse, death for any cause or last follow-up. OS was calculated from the date of initial treatment to the date of death for any cause or last follow-up. PFS and OS rates were estimated with their 95% CI using the Kaplan-Meier method. Analysis was based on the intention-to-treat, and was performed using SPSS statistical analysis software (version 12.0; SPSS Inc.). No comparison between the two cohorts of patients was planned. The end point database was updated in December 2014.
